# The Effectiveness of Lasers in Treatment of Oral Mucocele in Pediatric Patients: A Systematic Review

**DOI:** 10.3390/ma15072452

**Published:** 2022-03-26

**Authors:** Muhammad Shahrukh Khan Sadiq, Afsheen Maqsood, Fatema Akhter, Mohammad Khursheed Alam, Maria Shakoor Abbasi, Sheheryar Minallah, Fahim Vohra, Haytham Jamil Alswairki, Huda Abutayyem, Samir Mussallam, Naseer Ahmed

**Affiliations:** 1 Department of Oral Pathology, Bahria University Dental College, Karachi 07557, Pakistan; drshahrukhkhan1992@gmail.com (M.S.K.S.) afsheenmaqsood@gmail.com (A.M.); 2 Department of Surgical and Diagnostic Sciences, College of Dentistry, Dar Al Uloom University, Riyadh 13314, Saudi Arabia; mstfatima@dau.edu.sa; 3 Department of Preventive Dentistry, College of Dentistry, Jouf University, Sakaka 72345, Saudi Arabia; 4 Department of Prosthodontics, Altamash Institute of Dental Medicine, Karachi 75500, Pakistan; maria_shakoor@hotmail.com; 5 Dental Department, Jinnah Postgraduate Medical Centre, Karachi 75510, Pakistan; sherry_minallah@hotmail.com; 6 Department of Prosthetic Dental Science, College of Dentistry, King Saud University, Riyadh 11545, Saudi Arabia; fvohra@ksu.edu.sa; 7 School of Dental Sciences, Universiti Sains Malaysia, Kota Bharu 16150, Malaysia; hitham.swerki@gmail.com; 8 Center of Medical and Bio-Allied Health Sciences Research, Department of Clinical Sciences, College of Dentistry, Ajman University, Ajman P.O. Box 346, United Arab Emirates; h.abutayyem@ajman.ac.ae; 9 Private Clinic, Dubai P.O. Box 65882, United Arab Emirates; sameer444@hotmail.com; 10 Prosthodontics Unit, School of Dental Sciences, Health Campus, Universiti Sains Malaysia, Kota Bharu 16150, Malaysia

**Keywords:** dental laser, oral pathology, oral medicine, paediatric surgery, mucocele, pedodontics

## Abstract

The mucocele is the most common minor salivary gland associated disease of the oral cavity. It is also considered one of the most common biopsied oral lesions in pediatric patients. In recent years, extensive evidence has been published about the usage of lasers in treating mucoceles in pediatric patients. The aim of the present study was to assess the effectiveness of laser irradiation in the treatment of pediatric mucocele. An electronic search of databases (PubMed, Scopus, Web of Science and Google Scholar) was carried out in order to identify all relevant articles using a combination of the following keywords: “Pediatric”, “Oral”, “Mucocele”, “Dental”, “Oral Medicine”, “Soft Dental Lasers”, “Hard Dental Lasers”, and “Lasers,” for all case reports, case series, case-control and cohort studies published from 2007 to 2021. After limiting the search results, removing duplicate titles, and eligibility evaluation, 17 papers were enrolled in the study. Out of the total studies included, 10 articles were related to the diode (635 nm, 808 nm, 810 nm, and 980 nm), 5 to CO_2_ (10,600 nm), 3 to Er, Cr: YSGG (2780 nm), and 1 involving KTP lasers (532 nm). All studies indicated successful clinical results on mucocele excision with better intra- and post-operative indicators. The general characteristics and outcomes were summarized, and the quality of the studies was assessed using CARE guidelines in this systematic review. The reduction or absence of pain and bleeding, hemostasis, reduced operating time, minimal analgesic consumption, and an antibacterial effect were among the advantages of laser irradiation in the included studies. The laser has proven itself to play an effective role in the treatment of oral mucocele in paediatric patients.

## 1. Introduction

Oral mucoceles are non-neoplastic cystic lesions of major and minor salivary glands that commonly occur in the oral cavity [1]. Mucoceles are primarily classified under two headings: extravasation and retention types. Extravasation mucoceles are caused due to mechanical trauma in salivary ductal cells that culminates in the accumulation of mucin in the extracellular space. The retention type, which is a much rarer occurrence, is due to the result of mucin retention because of salivary ducts or acini obstruction [2]. The common site of mucoceles is the lower lip while other locations where mucoceles have been observed also include the ventral surface of the tongue, upper lip and floor of the mouth [3]. Although often asymptomatic, a mucocele, owing to its size and site of lesion can also cause a feeling of discomfort and pain, and subsequently compromise the functionality of the mouth. The traditional treatment for oral mucoceles of smaller size is surgical excision, while on the other hand marsupialization is employed for larger lesions [4].

The literature has reported a consistent number of cases of oral mucoceles in pediatric patients [5]. Although mucoceles are benign in nature, its presence can act as a major impediment to feeding and respiration [6]. The presence of pediatric mucoceles in infants is a frequent cause of concern and apprehension for parents [7]. In addition to an accurate and prompt diagnosis, the application of a treatment modality that effectively counters the limitations posed by dentistry in children is also essential. Dental treatment in pediatric patients, owing to their anxiety and fears, pose a challenge to clinical practitioners [8]. In addition, limitations in taking therapeutic actions may lead to general anesthesia instead of local ones, especially at younger ages, thereby further adding to the concern over complications caused by general anesthesia [7]. Similar to other soft tissue conditions in pediatric patients, an intervention that uses the minimum invasive approach reduces pain, requires only a short procedure time and is devoid of complications can provide more benefits and comfort to patients with oral mucoceles [8]. In this regard, technological advancements in dentistry have vitiated undesirable effects of such limitations, as the intraoral and extraoral use of dental lasers for performing surgical methods on oral soft tissue conditions has been met with successful results [9].

The last few decades have witnessed lasers as a helpful tool in the field of dentistry. Lasers have been utilized in various capacities that have subsequently revolutionized the diagnosis and treatment of oral lesions [10]. Primarily, the lasers operate through a laser-tissue interaction interface. The numerous advantages of lasers include their relative safe use, pain reduction, minimum invasion, shortening the time of surgical interventions, ability to achieve a precise cut, better accessibility and visibility of the surgeon due to bloodless field, analgesic effect and photobiomodulation promoting tissue healing and regeneration; these characteristics, therefore, make it a viable candidate for use in the excision of soft tissue lesions, including mucoceles [5,11]. Primarily, soft tissues lasers are of various types; diode, CO_2_ and YAG family lasers are the most commonly used, with each possessing unique benefits [11]. Diode lasers are poorly absorbed by hard dental tissues in contrast to the skin pigments melanin and hemoglobin, which readily absorb their irradiation with far greater thermal effects [12]. Usage of lasers, especially CO_2_ lasers, has also reportedly shown effective results due to the high absorption of its photons in water [13].

The null hypothesis of this study was that the laser is not the first choice or a better alternative to surgical excision of mucoceles, especially in paediatric patients. The application of lasers in treating mucoceles has been well reported in the literature, and it is therefore a requirement to critically analyze the quality of the studies in order to standardize and streamline the respective laser-related parameters of each laser type in the future. Therefore, the aim of the current systematic review was to assess the effectiveness of dental lasers in treating oral mucoceles in pediatric patients.

## 2. Materials and Methods

### 2.1. Focused Question

By using the Participants, Intervention, Control, Outcomes and study design protocol described in the Preferred Reporting Items for Systematic reviews and Metanalyses (PRISMA) guidelines [14], the focused question was constructed as follows: “Are dental lasers effective in the treatment of oral mucoceles in pediatric patients?”

### 2.2. Search Strategy

**Figure 1:**
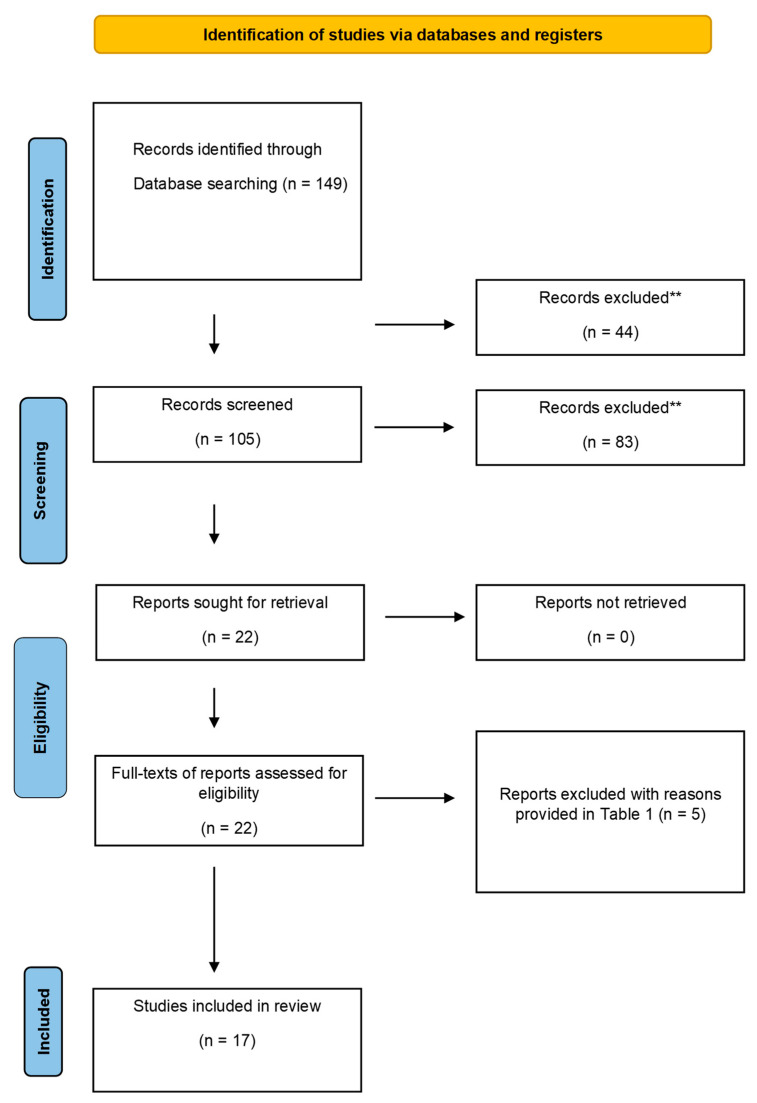
A PRISMA flow diagram for the search methodology used for the systematic review.

An electronic search was conducted via PubMed using a combination of free keywords and the Medical Subject Headings (MeSH) terms “oral”, “mucocele”, “dental laser” and “pediatric” for studies published from January 2007 to December 2021. A similar search was conducted via Google Scholar, PubMed, Medline, Scopus, Embase, and ISI Web of Knowledge. A secondary search was also performed by reading the reference lists of the articles that met the pre-determined inclusion criteria for additional studies relevant to this review. Two independent reviewers (MSKS and AM) screened titles and abstracts of all studies retrieved by the research strategy, and excluded the irrelevant studies. Full articles were retrieved for all potential studies and reviewed by the reviewers for inclusion afterwards. Moreover, the reference lists of the articles included were also read to find any additional articles that qualified the inclusion criteria. The literature search process is illustrated via PRISMA flow diagram in Figure 1.

### 2.3. Eligibility Criteria

The following predefined eligibility criteria were used to screen all identified studies.

The studies included in the systematic review met the following criteria:

1. Case reports
2. Case series
3. Case-control studies
4. Cohort studies
5. Studies reporting the use of dental lasers in the treatment of oral mucocele in pediatric patients
6. Studies in the English language

The following studies were excluded from the systematic review:

1. Clinical trials
2. Review articles (narrative and systematic)
3. Short communications
4. Letters to editors
5. Studies in languages other than English

### 2.4. Data Extraction

The data was extracted by investigators MSKS and AM using a special data collection form comprised of numerous headings: authors, year of study and country of the study, study design, total number of participants, age of participants, gender of participants, type of mucocele, size of mucocele, site of mucocele, type of laser, wavelength, energy, power, emission mode, device name, follow-up after treatment, and main outcomes. Any disagreements were solved by discussion. A third investigator NA validated the tables.

### 2.5. Quality Assessment of Studies

The quality of methodologies utilized in the studies was assessed by using the CARE guidelines (for CAseREports). The quality assessment was performed by two investigators—MSKS and NA—independently [15]. Any arising disagreements were solved by discussion. The quality of parameters associated with title, keywords, abstract, adequate introduction, timeline, patient description, clinical findings, diagnostic assessment, therapeutic intervention, follow-up/outcomes, discussion, patient perspective, funding information, and informed consent were assessed to determine the quality of each study. In the abstract section, background, case summary, and conclusion were assessed. The patient description was assessed according to the specific patient information, patient concern and symptoms, family and medical history, and relevant past interventions, if any. The diagnostic assessment was mentioned under clinical and histological types while therapeutic intervention comprised of numerous laser-associated parameters: wavelength, energy and power of laser used for the procedure. In follow/outcomes, clinican and patient assessed outcomes were reported, along with adverse events or complications, if any. In the discussion section, the strengths and limitations of the study results supported by evidence in the form of references and appropriate rationale for conclusion were evaluated to determine the quality of included studies.

## 3. Results

### 3.1. Results of Literature Search

The initial primary search resulted in a total of 149 studies. After exclusion of duplicates, abstracts, and titles, 105 studies were read to include the ones relevant to the specific review. After the exclusion of 83 irrelevant studies, the full texts of 22 studies were retrieved. After reading the full texts of these studies, 5 studies were excluded [16,17,18,19,20]. As shown in Table 1. A total of 17 studies fulfilled the criteria: case reports, case series, and a cohort study [21,22,23,24,25,26,27,28,29,30,31,32,33,34,35,36,37]. The inter-examiner reliability score (Kappa score) was calculated to be 0.82.

### 3.2. General Characteristics and Outcomes of Studies

The general characteristics and outcomes of the studies reviewed are summarized in Table 2. A total of seventeen studies were included in the systematic review, eleven of which were case reports (all prospective), five case series (two prospective and three retrospective), and a single cohort study (prospective) [21,22,23,24,25,26,27,28,29,30,31,32,33,34,35,36,37]. A total of 183 pediatric patients with mucoceles were treated with lasers. In addition to the pediatric patients, three studies included patients from other age groups as well [21,24,28]. The youngest patient in all of the studies was an infant of 3 months [29], while the oldest patient related to the pediatric group was 18 years-old [26]. All of the studies specified gender, except two [22,32]. In addition to pediatric oral mucocele, three studies utilized lasers in treating other oral conditions [32,33,34]. 2 studies also reported cases of surgical excision via scalpel, along with cases of laser treatment [24,26]. Mucoceles of a wide variety of sizes and diameter were treated, with the majority being of extravasation type [21,22,23,24,25,26,27,28,29,30,31,32,33,34,35,36,37], while only one study also reported mucoceles of the retention type [24]. The lower lip was the common site in all studies, but mucoceles at other locations were also reported that included buccal mucosa, tongue, floor of mouth, palate, and upper lip [24,26]. 10 studies used diode lasers of various wavelengths (635 nm, 808 nm, 810 nm and 980 nm) [25,27,28,29,30,31,33,35,36,37], 5 used CO_2_ laser (10, 600 nm) [21,22,24,26,32], 3 utilized Er,Cr:YSGG laser (2780 nm) [23,27,34], while 1 study also employed a KTP laser (532 nm) in the treatment [27]. Only two studies reported energy doses used during the procedure [32,37]. Power of the laser was reported by all studies, with 0.8 W [32] being the lowest and 8 W being the highest [21]. Follow-up period after treatment was mentioned in all studies [21,22,23,24,25,26,27,28,29,30,31,32,33,34,35,36,37]. At all wavelengths, laser treatment was effective and resulted in shorter procedural timings, better surgical site visualization, reduced bleeding, minimal discomfort, and reduced scarring with better healing and less post-operative pain [21,22,23,24,25,26,27,28,29,30,31,32,33,34,35,36,37]. 1 patient experienced local paresthesia at the operative site [21], 5 developed a recurrent mucocele [21,26,31], and 2 developed other complications [26]. In one study, excision was performed again using a diode laser on the recurrent mucocele, and a thermoplasticised splint was used as an adjunct to prevent nail biting and irritation of the lip from incisors [31]. One study used three different types of lasers; KTP and diode lasers offered the best bleeding control and a high cutting activity, though a precise and atraumatic cut was obtained by Er,Cr:YSGG, but all laser types involved met with good results [27].

### 3.3. Results of Quality Assessment

The quality assessment of each included study was evaluated in accordance with the CARE guidelines. The details of the quality analysis are provided in Table 3. All included 17 studies contained a suitable title and reported clinical findings adequately [21,22,23,24,25,26,27,28,29,30,31,32,33,34,35,36,37]. An appropriate abstract, relevant keywords, adequate introduction, sufficient patient description, and thorough discussion were included in most of the studies [21,22,23,24,25,26,27,28,29,30,31,32,33,34,35,36,37]. The diagnostic assessment (both clinically and histologically) was mentioned in 13 studies [21,23,24,25,26,27,29,30,31,33,35,36,37]. None of the studies reported a timeline of the dental procedure [21,22,23,24,25,26,27,28,29,30,31,32,33,34,35,36,37]. In the parameter of therapeutic intervention, type of laser, wavelength, and power were provided by all studies [21,22,23,24,25,26,27,28,29,30,31,32,33,34,35,36,37], while energy dose was mentioned in only two studies [32,37]. Patient perspectives of the treatment provided were described in none of the studies. Funding information was provided in only 2 studies [22,23,24,25,26,27,28,29,30,31,32,33,34,35,36]. Moreover, a total of 8 studies reported that the informed consent from patients were sought out [28,29,30,31,33,35,36,37]. 

## 4. Discussion

In recent years, lasers have found practical use in almost all aspects of dentistry. They have been used as a main or adjuvant tool for dealing with various pediatric oral soft tissue pathologies. The present study systematically reviewed the effectiveness of dental lasers in treating oral mucoceles in peadiatric patients.

The mucocele is one of the most common oral lesions. Different treatment modalities have been adopted in the past for its management, i.e., surgical excision, electrocautery, cryosurgery, marsupialization, and laser therapy. Conventional surgical excision with and without marsupialization remains the gold standard treatment protocol, as it pre-vents recurrence and is cost-effective. Despite these advantages, it might be associated with complications such as lip disfigurement, damage to adjacent salivary ducts, numbness, and scarring [38,39,40,41]. 

Lasers in dentistry began to gain popularity in the 1990s. They are used in dentistry as an adjunct or independent treatment tool. The main goal of using lasers over conventional treatment is to overcome the disadvantages of conventional treatment modalities [10]. Literature shows that laser therapy has limited postoperative bleeding, pain, complications, damage to the surrounding structures, shorter healing time, and relapse, as compared with scalpel excision [42]. Furthermore, studies have discovered that lasers in dentistry are highly tolerable and acceptable for children, which can improve treatment outcomes and make surgery and recovery easier [43,44]. This study evaluated 17 articles, where a total of 183 peadiatric patients with mucoceles were treated with different lasers [21,22,23,24,25,26,27,28,29,30,31,32,33,34,35,36,37]. The studies by Yagüe et al. and Wu et al. [24,26] reported cases of conventional surgical excision and laser, and they reported conclusively that laser therapy resulted in a more comfortable postoperative outcome for the patient, as compared to surgical excision.

The optimal type of laser for oral soft tissue surgeries is not clearly documented. The effectiveness of the Diode, CO_2_, and YAG families of lasers were assessed in this study. Out of the total included articles, 10 studies used diode lasers of various wavelengths [25,27,28,29,30,31,33,35,36,37]. Furthermore, a majority of the studies reported adequate postoperative healing with minimal or no scarring, no post-operative discomfort or pain, nor any other complication, and recurrence in the treated lesions. In addition, less procedure time, good surgical site visualization, and hemostasis were also achieved [25,27,28,29,30,33,35,36,37]. In this review, all the studies included a follow-up period that varied from 8 days to 1 year. However, the study by Chinta et al. [31] reported a case where reoccurrence was seen after 4 weeks of using a diode laser. For the prevention of future recurrences, excision was performed again, and a thermoplasticised splint was used as an adjunct to prevent nail biting and irritation of the lip from the incisors. No recurrence was observed after 6-month follow-up. Moreover, Romeo et al. [27] used KTP, Er, Cr: YSGG and diode lasers for removal of the mucocele and found that a diode laser offered optimum bleeding control and high cutting efficiency due to the higher affinity of a diode laser for hemoglobin.

Additionally, 05 studies assessed CO_2_ laser of various wavelengths (10–600 nm) and observed minimal pain or discomfort, no or reduced bleeding, and uneventful healing with a follow-up of 3 weeks to 1 month [21,22,24,26,32], even though Huang et al. and Wu et al. [21,26] reported that 2 of their cases developed a recurrent mucocele. Three of the studies utilized an Er,Cr:YSGG laser (2780 nm) and reported no discomfort or bleeding, little or no scarring, without recurrence [23,27,34]

Results of the reviewed studies indicate that the lasers have proven to be a safe and effective treatment modality for oral mucocele removal. It is well accepted by young patients because it is painless and has minimal or no postoperative complications. Therefore, it should be considered as the first choice or a better alternative to surgical excision, especially in paediatric patients.

Although the result of the included studies was favorable, there were certain limitations of this study. For instance, the included studies failed to provide considerable in-formation about timeline, patient perspective, and laser energy. A majority of the studies failed to report the recurrence rates, as most of the studies had a follow-up period of less than a month [21,22,24,25,26,27,30,32,33,34,36]. This review only included case reports and case series. Future studies should include clinical trials for comparison with conventional surgical procedures in different groups. Nonetheless, an increased number of patients should be reported for diversity; more importantly, long-term post-surgical follow-up periods and clinical correlation should be emphasized to predict the actual outcome of the laser radiation in the treatment of pediatric mucoceles.

## 5. Conclusions

Within the limitations of this review, it can be concluded that dental lasers of nu-merous types, wavelengths, and laser related parameters showed effectiveness in the treatment of mucoceles. An overwhelming majority of cases reported superior benefits that encompassed intra-operative and post-operative advantages to both clinician and patient. Due to the non-uniformity of laser-associated parameters, the overall quality of the literature was compromised. To establish uniform and standardized laser protocols for effective treatment of oral mucoceles in paediatric patients, studies of better quality are required.

## Figures and Tables

**Table 1:** Methodological list of studies excluded from this review and the reasons for exclusion (*n* = 5).

Study	Reason(s) for Exclusion
Silvia Pérez-García, 2005 [16]	Related to day case oral surgery
Choudhary, Deans & Moriarty, 2006 [17]	Related to pediatric nasolacrimal duct obstruction
Khosraviani et al., 2019 [18]	Systematic review
Ullrich, Malhotra & C. Patel, 2021 [19]	Narrative Review
Kato et al., 2021 [20]	Case report of non-pediatric patient

**Table 2:** General characteristics and outcomes of the included studies.

Author, Year of Study, Country	Study Design and No of Participants	Age of Participants (Years)	Gender of Participants	Type of Mucocele	Size(s) of Mucocele	Site(s) of Mucocele	Type of Laser	Wavelength	Power	Emission Mode	Follow Up after Treatment	Outcome
Huang et al., 2007 Taiwan/USA [21]	Retrospective case series, 82 participants	1–58 (Mean age = 20.8)	M = 45 F = 37	NA	>10 mm	Lower lip	CO_2_	NA	5–8 W	NA	1 to 3 week	No pain, bleeding, fast healing, however, parasthesia in 1, and recurrence in 2 patients found
Kato & Wijeyeweera, 2007 Sri Lanka [22]	Prospective case series, 76 participants	1–15	NA	NA		NA	CO_2_	10,600 nm	4 W	Continuous	1 to 3 weeks	Surgery wound healed in 3 weeks.
Boj et al., 2009 Spain [23]	Prospective Case report, 1 participant	9	F	Extravasation	4 mm diameter	Lower lip	Er,Cr:YSGG	2780 nm	1.5 W	NA	1 to 5 weeks, 1 year	Wound healed without scaring.
Yagüe-García et al., 2009 Spain [24]	Retrospective case series, 68 participants	6–65	M = 40 F = 28	Extravasation = 62. Retention = 06.	0.4–3 to 1–1.5 cm	Lower lip, togue, cheeks, palate	CO_2_	10,600 nm	5–7 W	NA	NA	Better esthetics, less bleeding, and parasthesia
Pedron et al., 2010 Brazil [25]	Prospective case reports, 2 participants	9–10	M = 2	Extravasation	2 cm 1 cm	Lower lip	Diode laser	808 nm 810 nm	2 W	Continuous	1 to 3 weeks,1 month	Reduced bleeding, discomfort, and scaring
Wu et al., 2011 Taiwan [26]	Retrospective case series, 64 participants	1–18 years (mean age = 12.3 ± 4.8 years)	M = 30 F = 34	Extravasation	NA	Lower lip = 57 Buccal mucosa = 02 Floor of mouth = 02	CO_2_	10, 600 nm	5 W	continuous	1 day 1 week 1 month	Laser treatment effective in 60 cases , whereas recurrence and scaringweres found in 4.
Romeo et al., 2013 Italy [27]	Prospective case series, 3 participants	12–14	M = 03 F = 00	Extravasation	0.5 to 1 cm	Lower lip	Er,Cr:YSGG, Diode, KTP.	2780 nm 808 nm 532 nm	2 W, 2 W, 1.5 W	NA	3 weeks	KTP and diode lasers offered adequate bleeding control, and healing
Pandey et al., 2013 India [28]	Prospective case reports, 2 participants	15–24	M = 01 F = 01	NA	1 × 1 cm in size	Lower lip	Diode	810 nm	1.3 W	NA	1 year	With diode lasers use reported minimum anesthesia, bleeding, no scarring and discomfort.
Paglia et al., 2015 Italy [29]	Prospective case report, 1 participant	3 months	M	Extravasation	1.5 cm in size	Lower lip	Diode	635–980 nm	1.7 W	Continous	2 to 24 weeks	No bleeding paresthesias, and recurrence was reported
Ramkumar et al., 2016 India [30]	Prospective case report, 1 participant	16	F	Extravasation	1 × 1 cm	Lower lip	Diode	940 nm	1.5 W	Continuous	1 to 45 days	Minimal anesthesia, and hemostasis, were observed.
Chinta et al., 2016 India [31]	Prospective Case report, 1 participant	9	M	Extravasation	4 mm diameter	Lower lip	Diode	810 nm	2 W	NA	4 to 24 weeks	Reoccurrence was seen in 4 weeks. Retreated tthan no reoccurrence observed after 6 months.
Hanna & Parker, 2016 England [32]	Prospective cohort study, 100 participant	4–15	NA	NA	0.5 × 0.5 cm	Lower lip	CO_2_	10,600 nm, 32 J	0.81 W	Continous	NA	No evidence of pain, scaring and recurrence of the mucocele.
Hegde et al., 2016 India [33]	Prospective case report, 1 participant	10	F	Extravasation	3 mm in diameter	Lower lip	Diode	810 nm	2 W	Continuous	8 days	Adequate excellent postoperative healing was reported without recurrence.
Kumar, Rehman & Chaturverdy, 2017 India [34]	Prospective case reports, 6 participant	9	M	Extravasation	NA	Lower lip	Er,Cr:YSGG	2780 nm	1.5 W	NA	NA	The wound healing was fast, and uneventful.
Vitale et al., 2018 Italy [35]	Prospective Case report, 1 participant	4 months	F	Extravasation	10 mm × 6 mm	Lower lip	Diode laser	810 nm	3 W	Continuous	2 weeks 4 months	The intraoral wound healed without complicatio, infection, and recurrence
Bagher et al., 2018 USA [36]	Prospective case reports, 3 participant	4–8	M = 1 F = 2	Extravasation	0.70 to 2 cm	Lower lip	Diode	980 nm	1.8 W	Continuous	1 month	Minimal scarring, no recurrence, and Discomfort.
Besbes et al., 2020 Tunisia [37]	Prospective case report, 1 participant	10	F	Extravasation	5 mm diameter	Lower lip	Diode	980 nm, 600 J	2 W	Continuous	2 weeks to 6 months.	Wound healed without complication, discomfort, and infection

NA, not available; F, female; M, male, cm, centimeter; mm, millimeter; CO_2_, carbon dioxide; Er, Cr: YSGG, erbium, chromium-doped yttrium, scandium, gallium and garnet; nm, nanometer; J, joule; W, wat.

**Table 3:** Results of the quality assessment of the included studies according to CARE guidelines.

Assessment Criteria	Huang et al., 2007 [21]	Kato et al., 2007 [22]	Boj et al., 2009 [23]	Yagüe-García et al., 2009 [24]	Pedron et al., 2010 [25]	Wu et al., 2011 [26]	Romeo et al., 2013 [27]	Pandey et al., 2013 [28]	Paglia et al., 2015 [29]	Ramkumar et al., 2016 [30]	Chinta et al., 2016 [31]	Hanna & Parker, 2016 [32]	Hegde et al., 2016 [33]	Kumar et al., 2017 [34]	Vitale et al., 2018 [35]	Bagher et al., 2018 [36]	Besbes et al., 2020 [37]
Title	Yes	Yes	Yes	Yes	Yes	Yes	Yes	Yes	Yes	Yes	Yes	Yes	Yes	Yes	Yes	Yes	Yes
Keywords	No	No	Yes	Yes	Yes	Yes	Yes	Yes	Yes	No	No	No	Yes	Yes	No	Yes	No
Abstract	Yes	Yes	Yes	Yes	Yes	Yes	Yes	Yes	Yes	Yes	No	Yes	Yes	Yes	No	Yes	Yes
Adequate introduction	Yes	Yes	No	Yes	No	Yes	No	Yes	Yes	No	No	Yes	Yes	Yes	Yes	Yes	Yes
Timeline	No	No	No	No	No	No	No	No	No	No	No	No	No	No	No	No	No
Patient description	No	No	Yes	No	Yes	No	Yes	Yes	Yes	No	Yes	No	Yes	Yes	Yes	Yes	Yes
Clinical findings	Yes	Yes	Yes	Yes	Yes	Yes	Yes	Yes	Yes	Yes	Yes	Yes	Yes	Yes	Yes	Yes	Yes
**Diagnostic assessment**																	
Clinical	Yes	No	Yes	Yes	Yes	Yes	Yes	No	Yes	Yes	Yes	No	Yes	No	Yes	Yes	Yes
Histological	Yes	No	Yes	Yes	Yes	Yes	Yes	No	Yes	Yes	Yes	No	Yes	No	Yes	Yes	Yes
**Therapeutic intervention**																	
Type of laser	Yes	Yes	Yes	Yes	Yes	Yes	Yes	Yes	Yes	Yes	Yes	Yes	Yes	Yes	Yes	Yes	Yes
Wavelength	Yes	Yes	Yes	Yes	Yes	Yes	Yes	Yes	Yes	Yes	Yes	Yes	Yes	Yes	Yes	Yes	Yes
Energy	No	No	No	No	No	No	No	No	No	No	No	Yes	No	No	No	No	Yes
Power	Yes	Yes	Yes	Yes	Yes	Yes	Yes	Yes	Yes	Yes	Yes	Yes	Yes	Yes	Yes	Yes	Yes
Follow-up/outcomes	Yes	Yes	No	Yes	Yes	Yes	Yes	Yes	Yes	No	Yes	Yes	Yes	Yes	Yes	Yes	Yes
Discussion	Yes	Yes	Yes	Yes	Yes	Yes	Yes	No	Yes	No	No	Yes	Yes	Yes	Yes	Yes	Yes
Patient perspective	No	No	No	No	No	No	No	No	No	No	No	No	No	No	No	No	No
Funding information	No	Yes	No	No	No	No	No	No	No	No	No	No	No	No	No	Yes	No
Informed consent	No	No	No	No	No	No	No	Yes	Yes	Yes	Yes	No	Yes	No	Yes	Yes	Yes

## Data Availability

The data presented in this study are available on request from the corresponding author.

## References

[1] Hayashida A.M., Zerbinatti D.C., Balducci I., Cabral L.A.G., Almeida J.D. (2010). Mucus extravasation and retention phenomena: A 24-year study. BMC Oral Health.

[2] Chi A.C., Lambert P.R., Richardson M.S., Neville B.W. (2011). Oral mucoceles: A clinicopathologic review of 1824 cases, including unusual variants. J. Oral Maxillofac. Surg..

[3] More C.B., Bhavsar K., Varma S., Tailor M. (2014). Oral mucocele: A clinical and histopathological study. J. Oral Maxillofac. Pathol..

[4] Cecconi D.R., Achilli A., Tarozzi M., Lodi G., Demarosi F., Sardella A., Carrassi A. (2010). Mucoceles of the oral cavity: A large case series (1994–2008) and a literature review. Med. Oral Patol. Oral Y Cir. Bucal.

[5] Kwok E.Y., Dovigi E.A., Eversole L.R., Dovigi A.J. (2015). Pediatric oral pathology: A retrospective survey of 4554 biopsies. Pediatr. Dent..

[6] Glickman A., Karlis V. (2016). Pediatric Benign Soft Tissue Oral and Maxillofacial Pathology. Oral Maxillofac. Surg. Clin. North Am..

[7] Ramazani N. (2016). Different aspects of general anesthesia in pediatric dentistry: A review. Iran. J. Pediatrics.

[8] Subcommittee C.A. (2015). American Academy of Pediatric Dentistry. Guideline on behavior guidance for the pediatric dental patient. Pediatr. Dent..

[9] Agoob Alfergany M., Alaijah F. (2020). Overview of the Clinical Benefits Using the Different Diode Laser Wavelengths in Treatment of the Mucocele: Clinical Cases Report Review. Photobiomodulation Photomed. Laser Surg..

[10] Luke A.M., Mathew S., Altawash M.M., Madan B.M. (2019). Lasers: A review with their applications in oral medicine. J. Lasers Med. Sci..

[11] Verma S.K., Maheshwari S., Singh R.K., Chaudhari P.K. (2012). Laser in dentistry: An innovative tool in modern dental practice. Natl. J. Maxillofac. Surg..

[12] Smeo K., Nasher R., Gutknecht N. (2018). Antibacterial effect of diode lasers in the treatment of peri-implantitis and their effects on implant surfaces: A literature review. Lasers Dent. Sci..

[13] Lai J.B., Poon C.Y. (2009). Treatment of ranula using carbon dioxide laser--case series report. Int. J. Oral Maxillofac. Surg..

[14] Moher D., Liberati A., Tetzlaff J., Altman D.G. (2009). Preferred reporting items for systematic reviews and meta-analyses: The PRISMA statement. Ann. Intern. Med..

[15] Gagnier J.J., Kienle G., Altman D.G., Moher D., Sox H., Riley D. (2013). The CARE guidelines: Consensus-based clinical case reporting guideline development. J. Med. Case Rep..

[16] Pérez-García S., Chaparro-Avendaño A.V., Delgado-Molina E., Berini-Aytés L., Gay-Escoda C. (2005). Day case oral surgery in pediatric patients during the year 2000 in the University of Barcelona Dental Clinic (Spain). Med. Oral Patol. Oral Y Cir. Bucal.

[17] Choudhary A., Deans J.A.J., Moriarty B.J. (2006). Modified laser DCR for paediatric nasolacrimal duct obstruction. Eye.

[18] Khosraviani F., Ehsani S., Fathi M., Saberi-Demneh A. (2019). Therapeutic effect of laser on pediatric oral soft tissue problems: A sys-tematic literature review. Lasers Med. Sci..

[19] Ullrich K., Malhotra R., Patel B.C. (2021). Dacryocystorhinostomy. Treasure Island.

[20] Kato R.B., Jácome-Santos H., Couto A.P., Abreu L.G., Mesquita R.A., de Oliveira Kato C.N. (2021). Management of Mucocele of the Glands of Blandin-Nuhn With a High-Intensity Laser: A Case Report. J. Lasers Med. Sci..

[21] Huang I.Y., Chen C.M., Kao Y.H., Worthington P. (2007). Treatment of mucocele of the lower lip with carbon dioxide laser. J. Oral Maxillofac. Surg..

[22] Kato J., Wijeyeweera R.L. (2007). The effect of CO_2_ laser irradiation on oral soft tissue problems in children in Sri Lanka. Photomed. Laser Surg..

[23] Boj J.R., Poirier C., Espasa E., Hernandez M., Espanya A. (2009). Lower lip mucocele treated with an erbium laser. Pediatr. Dent..

[24] Yagüe García J., EspañaTost A.J., BeriniAytés L., Gay Escoda C. (2009). Treatment of oral mucocele-scalpel versus CO_2_ laser. Med. Oral Patol. Oral Y Cir..

[25] Pedron I.G., Galletta V.C., Azevedo L.H., Corrêa L. (2010). Treatment of mucocele of the lower lip with diode laser in pediatric patients: Presentation of 2 clinical cases. Pediatr. Dent..

[26] Wu C.W., Kao Y.H., Chen C.M., Hsu H.J., Chen C.M., Huang I.Y. (2011). Mucoceles of the oral cavity in pediatric patients. Kaohsiung J. Med. Sci..

[27] Romeo U., Palaia G., Tenore G., Del Vecchio A., Nammour S. (2013). Excision of oral mucocele by different wavelength lasers. Indian J. Dent. Res..

[28] Pandey R., Pathakota K.R., Koppolu P., Bolla V. (2013). Treatment of mucocele with diode laser. J. Dent. Lasers.

[29] Paglia M., Crippa R., Ferrante F., Angiero F. (2015). Mucocele of the minor salivary glands in an infant: Treatment with diode laser. Eur. J. Paediatr. Dent..

[30] Ramkumar S., Ramkumar L., Malathi N., Suganya R. (2016). Excision of mucocele using diode laser in lower lip. Case Rep. Dent..

[31] Chinta M., Saisankar A.J., Birra C., Kanumuri P.K. (2016). Successful management of recurrent mucocele by diode laser and thermo-plasticised splint as an adjunctive therapy. Case Rep..

[32] Hanna R., Parker S. (2016). The advantages of carbon dioxide laser applications in paediatric oral surgery. A prospective cohort study. Lasers Med. Sci..

[33] Hegde R., Khare S., Saraf T., Shinde S. (2016). The Management Of Lower Lip Mucocele With Diode Laser In Pediatric Patient-An Alternative To The Surgical Approach. Indian J. Dent. Sci..

[34] Kumar G., Rehman F., Chaturvedy V. (2017). Soft tissue applications of Er, Cr: YSGG laser in pediatric dentistry. Int. J. Clin. Pediatric Dent..

[35] Vitale M.C., Sfondrini M.F., Croci G.A., Paulli M., Carbone L., Gandini P., Scribante A. (2018). Diode laser-assisted surgical therapy for early treatment of oral mucocele in a newborn patient: Case report and procedures checklist. Case Rep. Dent..

[36] Bagher S.M., Sulimany A.M., Kaplan M., Loo C.Y. (2018). Treating mucocele in pediatric patients using a diode laser: Three case reports. Dent. J..

[37] Besbes A., Elelmi Y., Khanfir F., Belgacem R., Ghedira H. (2020). Recurrent Oral Mucocele Management with Diode Laser. Case Rep. Dent..

[38] Bowers E.M.R., Schaitkin B. (2021). Management of Mucoceles, Sialoceles, and Ranulas. Otolaryngol. Clin. N. Am..

[39] Mínguez-Martinez I., Bonet-Coloma C., Ata-Ali-Mahmud J., Carrillo-García C., Peñarrocha-Diago M., Peñarrocha-Diago M. (2010). Clinical characteristics, treatment, and evolution of 89 mu-coceles in children. J. Oral Maxillofac. Surg..

[40] Karthiga Kannan S., Eugenia Sherubin J., Priya M.S., Mouetaz Kheirallah Salama M.H. (2016). Cystic oral lesions of salivary gland origin in children: Case series—An Observational study. Majmaah J. Health Sci..

[41] Bahadure R.N., Fulzele P., Thosar N., Badole G., Baliga S. (2012). Conventional surgical treatment of oral mucocele: A series of 23 cases. Eur. J. Paediatr. Dent..

[42] Ata-Ali J., Carrillo C., Bonet C., Balaguer J., Peñarrocha M., Peñarrocha M. (2010). Oral mucocele: Review of the literature. J. Clin. Exp. Dent..

[43] Angiero F., Ugolini A., Cattoni F., Bova F., Blasi S., Gallo F., Cossellu G., Gherlone E. (2020). Evaluation of bradykinin, VEGF, and EGF biomarkers in gingival crevicular fluid and comparison of Photo Biomodulation with conventional techniques in periodontitis: A split-mouth randomized clinical trial. Lasers Med. Sci..

[44] Polizzi E., Tetè G., Targa C., Salviato B., Ferrini F., Gastaldi G. (2020). Evaluation of the Effectiveness of the Use of the Diode Laser in the Reduction of the Volume of the Edematous Gingival Tissue after Causal Therapy. Int. J. Environ. Res. Public Health.

